# Changes in psychiatric admissions in the first year of COVID-19 in Ontario, Canada

**DOI:** 10.1186/s13033-025-00674-w

**Published:** 2025-06-03

**Authors:** Gustavo S. Betini, Dorothy Yu, Edgardo Perez, Jitender Sareen, Christopher M. Perlman, John P. Hirdes

**Affiliations:** 1 School of Public Health Sciences, University of Waterloo, Waterloo, ON Canada; 2 Department of Psychiatry, Max Rady College of Medicine, University of Manitoba, Winnipeg, MB Canada; 3 Faculty of Medicine, University of Ottawa, Ottawa, ON Canada

**Keywords:** Mental health services, Pandemic, Psychiatric symptoms, Involuntary admission, Substance use, InterRAI

## Abstract

**Background:**

Several studies showed strong evidence that the COVID-19 pandemic disrupted mental health service use, with changes in emergency department visits, and psychiatric hospital admissions. It is not clear, however, whether the pandemic caused an increase or decrease in use of services for people with different diagnoses and symptoms.

**Methods:**

We used data from all individuals admitted to psychiatric units in Ontario, Canada (259,620 individuals) from January 1st 2015 to December 31st, 2020 and compared the number of admissions, length of stay, symptoms, and clinical characteristics of this population in 2020 to the average of those who were admitted between 2015 and 2019.

**Results:**

Total number of admissions declined sharply (44%) during the first lockdown period but returned to pre-pandemic levels within about 2 months. This trend, however, was not observed for all types of mental health problems. Admissions for symptoms such as risk of harm to others and addictions were consistently higher after the first wave in May 2020 compared to the same month in the previous 5 years, while symptoms such as social withdrawal, and depression were consistently lower.

**Conclusion:**

Taken together, these results suggest that the impact of the pandemic on the use of mental health services were symptom-specific, which is likely a result of the heterogeneity of mental health problems within this population. This variation in the changes in psychiatry admissions for patients with different clinical profiles should be considered when preparing for future service interruptions.

**Supplementary Information:**

The online version contains supplementary material available at 10.1186/s13033-025-00674-w.

## Background

The COVID-19 pandemic caused a great disruption in health care systems around the world with declines in the number of people admitted in hospitals for non-COVID-19 related problems [1–9], delay of services [1, 3], and even a decline in planned and life-saving surgeries [7]. Across Canada, the number of people admitted to hospitals for non-COVID-19 related problems dropped by 50% during the first COVID-19 wave beginning in March 2020. There was also a 22% decrease in people transferred to hospitals compared to the same period in previous years. This change in behaviour has the potential to delay treatment and worse patient conditions [10].

The global pattern of mental health-related emergency and hospital visits was quite varied when compared to similar periods in the pre-COVID era. While many studies have shown significant decreases in the number emergency department visits for mental health problems and/or psychiatry admissions around the world [9, 11–26], some showed no change [27–31] or even an increase in visits and/or admissions [32–35]. Inpatient occupancy rates and length of stay showed similar patterns with increases [20, 27], and decreases [27] reported in the published literature.

Across these findings there are certainly methodological differences in how mental health of the general population was analyzed. For instance, public health COVID-19 policies varied across jurisdictions, which could have restricted access to services at different point in time (e.g., some countries experienced longer and more restricted lockdowns than others). Therefore, the mental health impact of the pandemic might differ depending on the timing of the pandemic and the public health measurements used to contain the spread of the virus. In Ontario, Canada during the first wave of the pandemic there were restrictions to access hospital care and a reduction in outpatient and primary care visits with a 30% decline in psychiatry admissions and 37% decline in emergency department visits for mental health problems in April 2020 (around 2 months after the first cases of COVID-19 in Canada) compared to 2019 [23–24].

With this change in visit and admission rates in mind, the purpose of this study is to understand whether there were clinical subpopulations that were differentially affected by the pandemic in their use of mental health services. To achieve this goal, we investigated temporal patterns in the reasons for admission, mental health symptoms, and diagnoses of psychiatric admissions in Ontario in 2020 compared with the average of the previous 5 years (2015–2019) as well as total volume of admissions and length of stay.

## Methods

### Study design and setting

We used a retrospective trend analysis of hospital admission records for all individuals 18 years and older who were admitted to hospitals for psychiatric reasons in Ontario, Canada from January 1st, 2015 to December 31st, 2020. We used 5 years of data (number of admissions, length of stay, reasons for admission, mental health symptoms, and diagnoses of psychiatric admissions) to provide a stable estimate of admission profiles prior to the pandemic.

### Data source

Data were obtained from Ontario Mental Health Reporting System (OMHRS), managed by the Canadian Institute for Health Information [36]. OMHRS contains information of all individuals aged 18 years and older who were admitted to an impatient mental health hospital in Ontario (Table 1). Data stored in OMHRS are based on the RAI-Mental Health, which is a comprehensive, person-centered, standardized mental health assessment instrument designed to evaluate the individual’s needs, challenges, and strengths to support care planning across a variety of domains [37, 38]. These assessments are conducted by trained mental health care professionals who are familiar with the patient (usually nurses) at day 3 of admission, discharge, or after 90 days in hospital, or when there is a clinically significant change requiring a modification of the care plan. Patients who are in hospital for less than 3 days are assessed with a subset of RAI-MH items rather than the full set of items in the RAI-MH instrument. We, therefore, excluded those short stay patients from the present analyses. To complete the assessment, clinicians use all sources of information available including interviews with the person and members of the support network, their own observations of the person, chart reviews, and discussion with other health practitioners involved in the person’s care. There are over 400 items in each assessment, including sociodemographic characteristics, and provisional diagnoses. Provisional diagnosis is a diagnosis made at admission based on the chapter headings of the DSM-V (e.g., Depressive Disorder). A more precise diagnosis is available at admission (e.g., Major Depressive Disorder). Social relations, clinical, and functional status are measured with different clinical summary scales.

**Table 1:** Distribution of clinical and demographic variables by COVID period

Variable		Pre-COVID-19		COVID-19	
Gender	Female	103,136	47.6%	19,832	46.2%
	Male	113,471	52.4%	23,052	53.7%
Age	18–25	38,678	17.8%	7,622	17.8%
	25–45	87,153	40.2%	18,376	42.8%
	45–65	65,080	30.0%	11,799	27.5%
	65–80	20,187	9.3%	4,090	9.5%
	80	5,604	2.6%	1,026	2.4%
Positive Symptoms Scale	0	105,199	48.5%	19,404	45.2%
	1–2	23,386	10.8%	4,757	11.1%
	3–5	40,246	18.6%	8,579	20.0.%
	6–12	47,873	22.1%	10,176	23.7%
Depression Severity Index	0	62,626	28.9%	13,859	32.3%
	1–3	68,124	31.4%	13,676	31.9%
	4–7	52,267	24.1%	9,729	22.7%
	8–15	33,687	15.5%	5,652	13.2%
Mania Scale	0	92,433	42.6%	17,843	41.6%
	1–3	52,992	24.4%	10,196	23.8%
	4–8	46,991	21.7%	9,693	22.6%
	9–20	24,288	11.2%	5,184	12.1%
Social Withdrawal Scale	0	96,864	44.7%	21,482	50.1%
	1–4	52,838	24.4%	10,431	24.3%
	5–8	38,467	17.7%	6,489	15.1%
	9–12	28,535	13.2%	4,514	10.5%
Risk of Harm to Others Scale	0	61,275	28.3%	11,836	27.6%
	1–2	86,457	39.9%	16,130	37.6%
	3–4	37,486	17.3%	7,746	18.0%
	5–6	31,486	14.5%	7,204	16.8%
Severity of self-harm scale	0	44,665	20.6%	9,553	22.3%
	1–2	77,791	35.9%	15,563	36.3%
	3–4	37,290	17.2%	6,688	15.6%
	5–6	56,958	26.3%	11,112	25.9%
Police Involvement	0	128,853	59.5%	24,052	56.0%
	1	87,851	40.5%	18,864	44.0%
Involuntary Admission	0	178,554	82.4%	34,289	79.9%
	1	38,150	17.6%	8,627	20.1%
Substance	0	159,271	73.5%	30,696	71.5%
	1	57,433	26.5%	12,220	28.5%
Harm to self	0	107,026	49.4%	21,405	49.9%
	1	109,678	50.6%	21,511	50.1%
Harm to Others	0	171,399	79.1%	32,925	76.7%
	1	45,305	20.9%	9,991	23.3%
Self care	0	126,574	58.4%	25,057	58.4%
	1	90,130	41.6%	17,859	41.6%
Total		216,704		42,916	259,620

The Pre-COVID-19 period refers to individuals admitted to psychiatric units in Ontario from January 1st 2015 to December 31st, 2019. COVID-19 refers to January 1st 2020 to December 31st, 2020

### Independent variables

We investigated 12 independent variables: five reasons for admission (harm to others, police involvement, involuntary admission, harm to self, and self care), one diagnosis (substance use and addictions), and six scales indicating symptom severity (Risk of Harm to Others Scale, Positive Symptoms Scale, Mania Scale, Severity of Self-Harm scale, Depression Severity Index, and Social Withdrawal Scale; please see Table S1 and S2 for a more detailed explanation of the independent variables). Most of these scales have been validated with Cronbach’s Alpha ranging from 0.70 to 0.89; [38–43]). Substance use and addiction refers to any substance use disorder according to the DSM-IV/V [44]. We used provisional categories of diagnoses at admission because a substantial proportion of episodes do not have a specific DSM-coded psychiatric diagnosis until discharge. Admissions in 2015 used the general categories of the fourth edition of the DSM [45] for provisional diagnosis and admissions between 2016 and 2020 used the categories specified in fifth edition [46]. Diagnoses were considered present if they were identified as the first, second, or third most important diagnosis contributing to the person’s hospitalization.

### Statistical analysis

Two issues were of interest with respect to changes in the admissions: absolute volumes of admissions of persons with different clinical characteristics and relative changes in the composition of clinical characteristics of persons admitted. In order to obtain a stable estimate of usual pre-pandemic admission patterns, we computed 5-year averages of monthly volumes of patients admitted with specific clinical characteristics. We compared these to the volumes for each month in the first year of the pandemic and investigated whether subtypes of patients were more or less likely to be admitted during the pandemic with logistic regression models. The period between January 1st, 2015 and December 31st, 2019 (pre-COVID-19) was coded as 0 and the period between January 1st to December 31st, 2020 (COVID-19) was coded as 1. We then used clinical characteristics as independent variables in the models to determine whether those attributes were more or less likely to be represented in the COVID-19 admissions.

Because there was a strong correlation between some of the variables we wanted to investigate, we built three alternative models to deal with issues of multicollinearity (Table S2). We explored models that considered three aspects of the person’s clinical profile: (a) the symptoms present at admission; (b) the purported reason for admission; and (c) the provision psychiatric diagnosis at admission. Our aim was not to develop a single definitive predictive model of admission patterns, but to test whether there were clinically important changes in admission profiles from alternative perspectives. Model A included all scales plus two reasons for admissions; model B included a reduced set of scales with all reasons for admissions; and model C includes only diagnosis (Table S2). All three models had age and sex as covariates. We reported any significant change in 12 independent variables (harm to self, harm to others, self care, problems with substance use and/or police involvement and involuntary admission, Risk of Harm to Others Scale, Positive Symptoms Scale, Mania Scale, Severity of Self-Harm Scale, Depression Severity Index, and Social Withdrawal Scale) if they were significant in at least one of the three models (Table S2). We conducted one logistic regression for each month of the year (Tables S3 and S4).

## Results

**Fig. 1:**
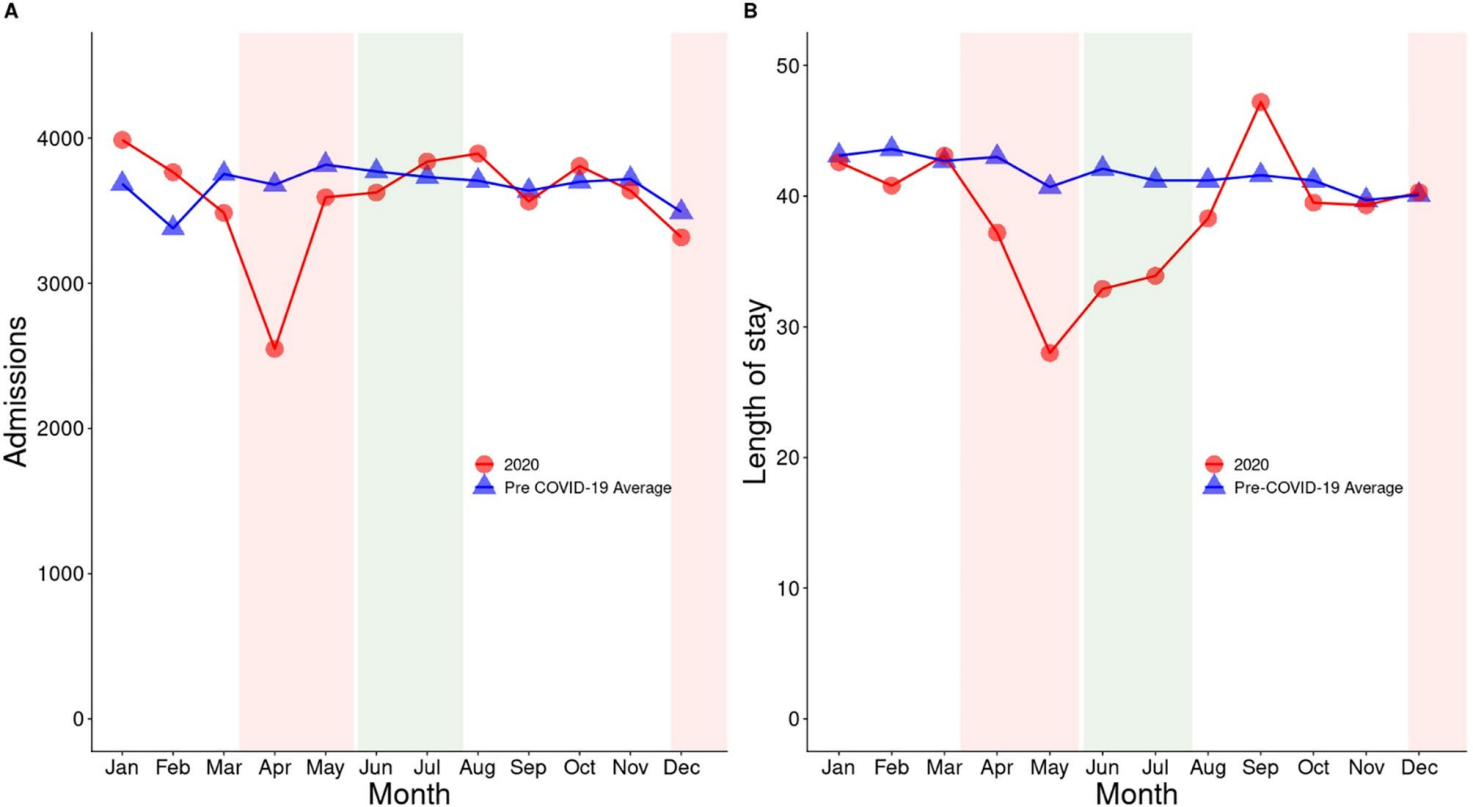
Trends in total number of psychiatric hospital/unit admissions (**A**) and length of stay (**B**) by time period. COVID-19 period (2020) is represented by red line with dots and 2014-19 Pre-COVID-19 period is represented by blue line with triangles. Shaded red areas are periods of lockdown and shaded green area is the period of re-opening. Values in (**A**) are presented in Table S4

Among 259,620 participants, we observed a sharp decline in total number of admissions in psychiatry hospitals in Ontario in April 2020, around two months after the first cases of COVID-19 were reported in Canada (1,130 fewer admissions than expected or a 44% decline; Table S4, Fig. 1A and Figure S1). In addition, there was a substantially shorter length of stay in May 2020 compared to April and May, respectively, in the previous 5 years (13 days or a 46% decline; Table S4 and Fig. 1B). Although the number of admissions after April 2020 fluctuated around the average of the previous 5 years (Fig. 1A), length of stay slowly increased from June to September, reaching a peak in September with 14% (~ 5 days, Table S4) longer stays than the previous years (Fig. 1B).

**Fig. 2:**
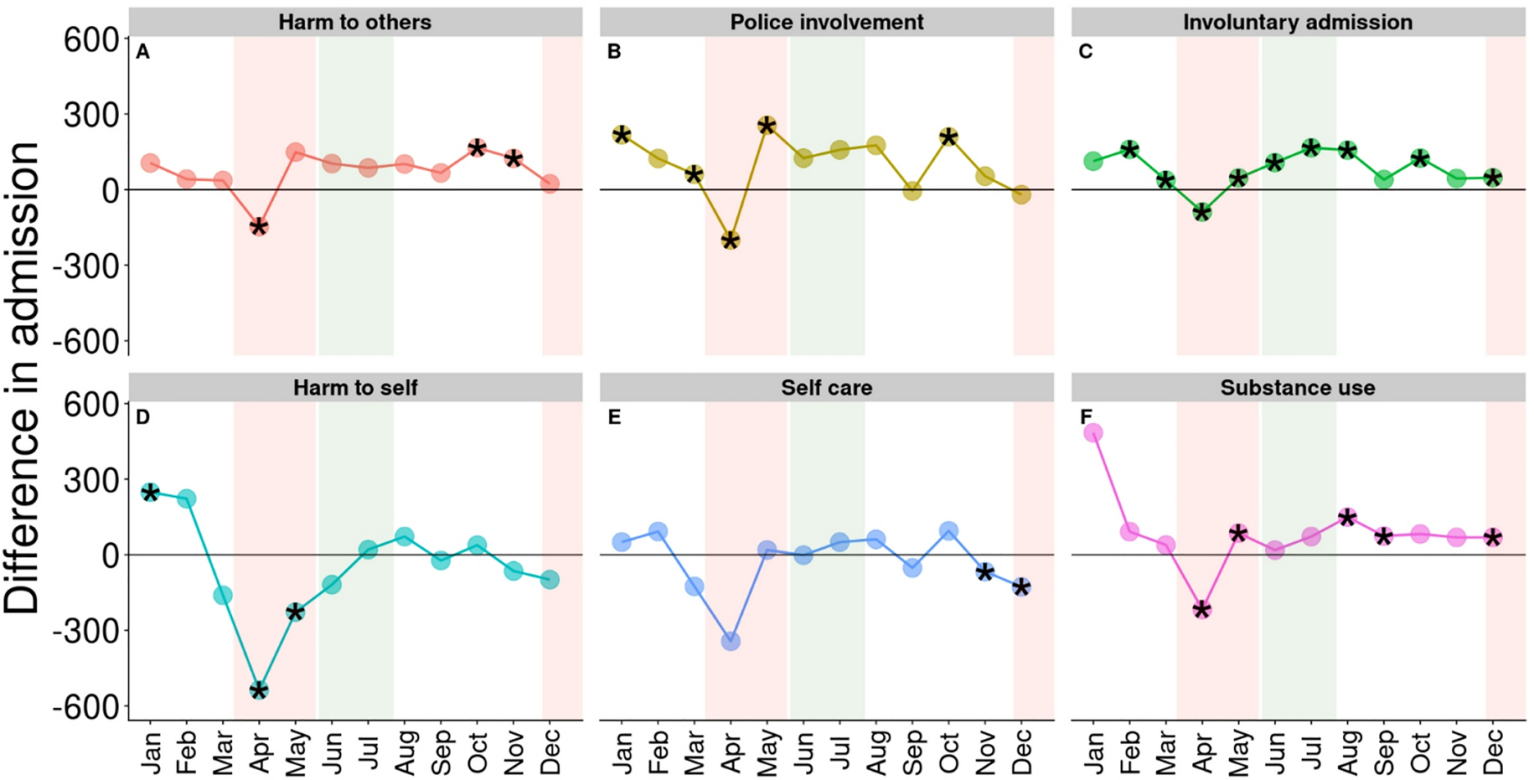
Difference in admission volumes for the PRE-COVID-19 and COVID-19 periods by 6 types of reason for admission. Horizontal black lines signify no change. Stars indicate changes that were statistically significant according to at least one of the three statistical models used. Shaded red areas are periods of lockdown (from mid March to mid May and from end of November to end of December) and shaded green area is the period of re-opening (from mid May to end of July)

**Fig. 3:**
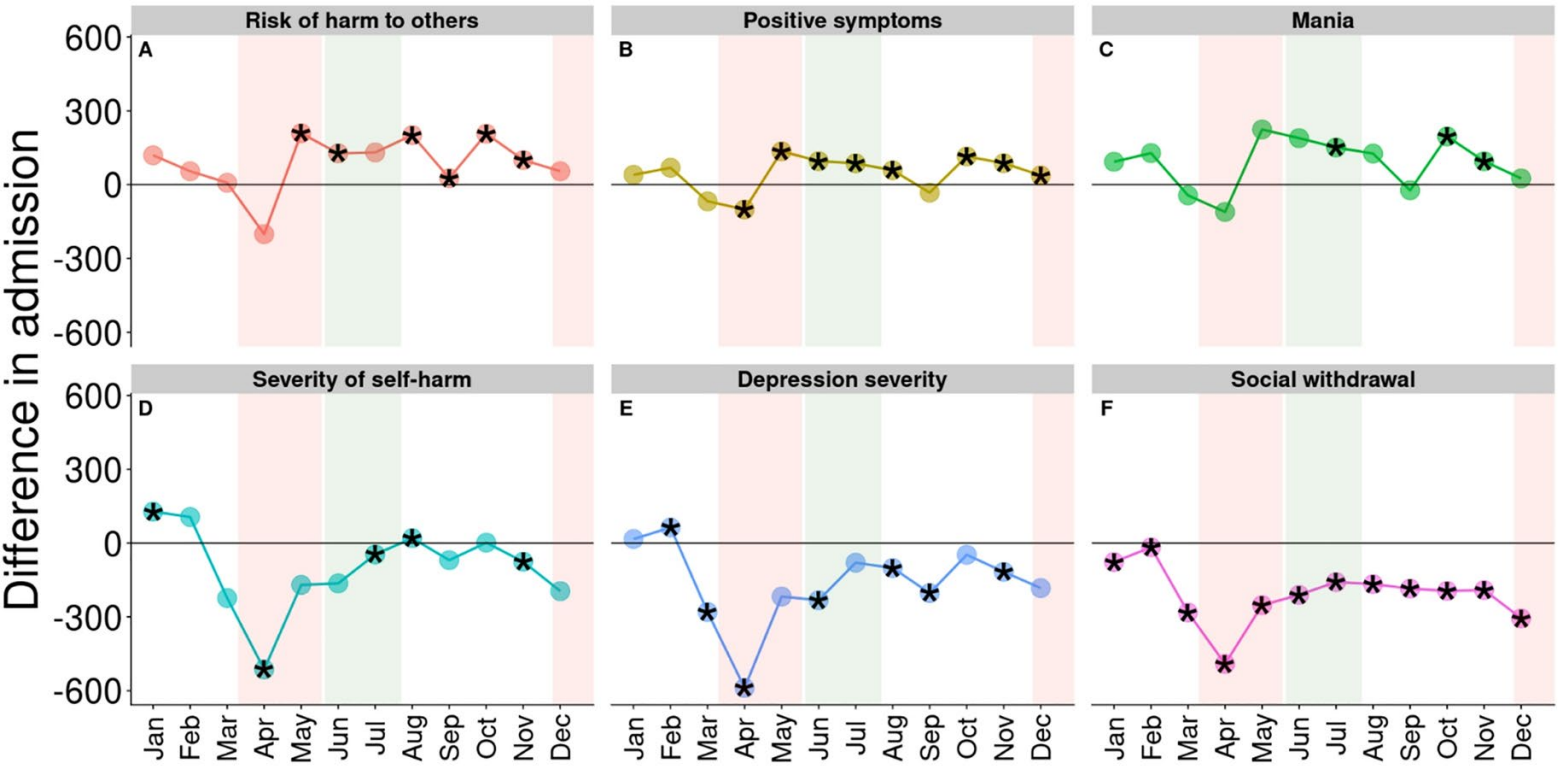
Difference in admission volumes for the PRE-COVID-19 and COVID-19 periods by 6 clinical summary scales. Horizontal black lines signify no change. Stars indicate changes that were statistically significant according to at least one of the three statistical models used. Shaded red areas are periods of lockdown (from mid March to mid May and from end of November to end of December) and shaded green area is the period of re-opening (from mid May to end of July)

There was also a general decline in the number of admissions associated with the 12 indicators of interest (Figs. 2 and 3). However, not all declines were statistically significant, such as admissions due to lack of self care (Fig. 2E), symptoms of harm to others, and mania (Fig. 3A and C, respectively). The greatest decline in reasons for admission among all 12 indicators analyzed was observed for harm to self (536 fewer admissions or a 41% decline) while the greatest declines in symptoms were observed for depression (589 fewer admissions or a 69% decline; Fig. 3E) followed by severity of self-harm (514 fewer admissions or a 50% decline; Fig. 3D). Social withdrawal had the biggest change (491 fewer admissions or a 76% decline; Fig. 3F).

Seven indicators showed a significant increase in at least 2 months after April 2020 compared to the previous 5 years: admissions for harm to others; police involvement; involuntary admissions; diagnosis of substance use (Fig. 2A, B, C, and F, respectively); risk of harm to others; positive symptoms; and mania (Fig. 3A, B, and C, respectively). Involuntary admissions and positive symptoms were consistently higher from May to December (6 and 7 months out of 8 analyzed, respectively). In contrast, severe of self harm, depression severity, and social withdrawal (Fig. 3D, E, and F, respectively) were significantly less common after April 2020 compared to the same months between 2015 and 2019. Admissions of patients with social withdrawal symptoms were consistently lower in all of 2020 (Fig. 3F).

## Discussion

There was a dramatic change in admission volumes and profiles among all inpatient psychiatric admissions in Ontario, Canada during the first year of the pandemic (2020) compared to the previous 5-year average. Across 259,620 individuals, there was an overall significant decrease in number of admissions early in 2020 [15, 17, 23, 24] and length of stay [20, 27]. On the other hand, we found that the volume of admissions tended to quickly return to pre-pandemic levels [23, 24, 26, 30]. However, our study found that not all clinical characteristics followed this pattern of return to historical levels. After the first lockdown in April 2020, we observed a significant increase in admissions compared to the five previous years for those issues related to externalizing symptoms (e.g., risk of harm to others, and positive symptoms) while there was a decrease in number of admissions for reasons related to internalizing symptoms (e.g., social withdrawal and depression). This is consistent with the findings that people with symptoms that interfere with social life, such as depression, might be more sensitive to disruptions to their social environment, as well as limited access or fewer opportunities to care [47]. Thus, our results suggest that attending only to changes in the total number of psychiatric admissions can miss important variations in the patient profiles of those using mental health services during the pandemic. Persons admitted to inpatient psychiatry do not comprise a homogeneous population. This diversity should be taken into account when attempting to understand differential patterns in access to or use of mental health services.

Our results might help to unveil some of the reasons why the pandemic caused significant changes in mental health services around the world. To explain the widespread decrease in number of emergency department visits for mental health problems and psychiatry admissions, it has been hypothesized that people were more reluctant to go to hospitals because of fear of contracting the virus or to avoid disrupting services that could be used for COVID-19 patients (the ‘*hospital avoidance*’ hypothesis; [14, 16, 17, 27, 28]). It is also possible that hospitals might have admitted only severe cases to make room for COVID-19 patients or to avoid cross contamination within hospital (the ‘*hospital constraint*’ hypothesis; [27, 28, 34]). Our results suggest that these hypotheses might be specific to the type of mental health problems that individuals experienced during the pandemic. For example, individuals with internalizing symptoms, who were less frequently admitted during the first wave of the pandemic in our study, may have been more likely to avoid hospitals during the lockdown, giving support to the hospital avoidance hypothesis. Indeed, there is evidence that individuals with internalizing symptoms had higher levels of depression during the pandemic [47], which could explain the increase in social isolation and, therefore, decrease in help-seeking behaviour. Conversely, the number of admissions may have increased for individuals with externalizing symptoms because their symptoms were more likely to be seen as serious and/or dangerous to others resulting in police involvement and/or involuntary admissions. It is rare that such cases are denied hospitalization by health care workers, giving support for the hospital constraint hypothesis. Other studies have found similar patterns during the first months of the pandemic, with increases in admissions under the Mental Health Act in United Kingdom [13, 21] increases in involuntary admissions in Sweden [12], Germany [31], and Israel [30], increase in substance use in Spain [15], Germany [31], US [27], and United Kingdom [9], and increase in admission for psychotic disorders in Canada [26]. Thus, the kind of mental health problem a person experience might be particularly pertinent in determining who is going to be admitted to psychiatry units during periods of crisis and service disruptions.

The shorter length of stay we observed for several months in 2020 (from April to August 2020) compared to the 2015–2019 period seems to also give support to the hospital constraint hypothesis. This is in line with reports of more restrict criteria for admission of psychiatry patients during lockdowns [48]. Interestingly, length of stay took much longer to return to similar pre-pandemic levels compared to the total number of admissions. It is possible that hospital staff had a lower threshold for discharge, leaving only the most severe cases admitted, either to conserve resources for COVID-19 cases, to avoid overcrowding [49], and/or because they were trying to avoid cross contamination between hospitals wards.

### Strengths and limitations

Our study uses population level data from all individuals 18 years and older admitted to all psychiatry units in Ontario from 2015 to 2020. This dataset contains important information about clinical characteristics and symptoms of patients that are assessed with a reliable instrument (the RAI-MH). However, we did not examine short stay hospitalizations, general emergency department visits, use of community services, and visits to family physicians. Perhaps some individuals who would have been admitted to hospital pre-pandemic were redirected or pursued care in these other settings. Although these services may have been unavailable during the first lockdown, many returned or were offered in a virtual format in the following months. Our study focuses on cases that were considered serious enough to require hospitalization of three days or more and might not be generalizable to mental health problems that are treated in the community.

## Conclusion

We examined changes in the volumes and clinical profiles of patients admitted to psychiatric hospitals in Ontario, Canada during the first year of the COVID-19 pandemic (2020) compared with the pre-pandemic 5-year average (2015–2019). Our results suggest that the impact of the pandemic varied depending on the characteristics of the patients, including reasons for admission, mental health symptoms, and diagnose, with an increase in admissions for those with externalizing symptoms while admissions for internalizing symptoms decreased. We suggest that these results are likely a consequence of the heterogeneity of mental health problems within this population, which public health officials should take into account when preparing for future service interruptions.

## Electronic supplementary material

Below is the link to the electronic supplementary material.

Supplementary Material 1

## Data Availability

Access to the Ontario Mental Health Reporting System data used for these analyses can be achieved through the Canadian Institute for Health Information. The authors do not have permission to transmit data to third parties.
