# Supplementary material for: Changes in psychiatric admissions in the first year of COVID-19 in Ontario, Canada
Source: Int J Ment Health Syst. 2025 Jun 3;19:18. doi: 10.1186/s13033-025-00674-w (PMC12131648; doi:10.1186/s13033-025-00674-w)
Supplement: Supplementary file 1 — Supplementary Material 1 [file 13033_2025_674_MOESM1_ESM.docx]

**Changes in psychiatric admissions in the first year of COVID-19 in Ontario, Canada**

Gustavo S. Betini^1^*, Dorothy Yu^2^, Edgardo Perez^3^, Jitender Sareen^2^, Christopher M. Perlman^1^, John P. Hirdes^1^

^1^ *School of Public Health Sciences, University of Waterloo, Waterloo, ON, Canada*

^2^ *Max Rady College of Medicine, Department of Psychiatry, University of Manitoba, MB, Canada*

^3^ *Faculty of Medicine, University of Ottawa, ON, Canada*

**Corresponding author:** [**gbetini@uwaterloo.ca**](mailto:gbetini@uwaterloo.ca)

**Figure S1**. Daily number of positive COVID-19 cases in Ontario, Canada. Shaded red areas are periods of lock-down and shaded green area is the period of re-opening. Data for daily number of COVID-19 cases in Ontario in 2020 were obtained from the publicly available source The COVID-19 Canada Open Data Working Group: opencovid.ca (Berry I, O’Neill M, Sturrock SL, Wright JE, Acharya K, Brankston G, Harish V, Kornas K, Maani N, Naganathan T. A sub-national real-time epidemiological and vaccination database for the COVID-19 pandemic in Canada. *Scientific Data* (2021) 8:173)

**Table S1.** Scales used to investigate changes in admission for different symptoms and clinical profile of participants.

| **InterRAI scale** | **Components** |
| --- | --- |
| **Risk of Harm to Others Scale** | Violence/extreme behaviour; violent ideation; intimidation/threats |
| **Positive Symptoms Scale** | Hallucinations; delusions; abnormal thought process |
| **Depression Severity Index** | Sadness; pained facial expressions; negative statements; self-deprecation; guilt/shame; hopelessness |
| **The Severity of Self-Harm Scale** | Self-harm ideation; suicide attempts; suicide plan |
| **The Social Withdrawal Scale** | Lack of motivation; reduced interaction; decreased energy; flat or blunted affect; anhedonia; loss of interest |
| **Mania scale** | Inflated self-worth; hyperarousal; irritability; increased sociability/hypersexuality; pressured speech; labile affect; sleep problems/ hypomania |
| **Cognitive Performance Scale** | Daily decision making; short-term memory; making self understood; performance in eating |
| **Self-Care index** | Insight into mental health, decreased energy, abnormal thought process, and expression (i.e. making self  understood) |
| **Aggressive Behaviour Scale** | Verbal and physical abuse; socially inappropriate and/or disruptive; resists care |

**Table S2.** Items from the RAI-MH instruments used in the analysis. Different logistic regression models (Model A, B, and C) were used because of high correlation between some items. Results from all three statistical models for all 12 months are in Table S4.

|  | **Model A** | **Model B** | **Model C** |
| --- | --- | --- | --- |
| **Demographics** |  |  |  |
| Age | x | x | x |
| Gender | x | x | x |
|  |  |  |  |
| **Scales** |  |  |  |
| Positive Symptoms Scale | x | x |  |
| Cognitive Performance Scale | x | x |  |
| Depression Severity Index | x | x |  |
| Aggressive Behaviour Scale | x | x |  |
| Mania | x | x |  |
| Social Withdrawal Scale | x | x |  |
| Risk of Harm to Others Scale | x |  |  |
| Severity of Self-Harm scale | x |  |  |
| Self-Care Index | x |  |  |
|  |  |  |  |
| **Reasons for admission** |  |  |  |
| Police Involvement | x | x |  |
| Involuntary Admission | x | x |  |
| Harm to Self |  | x |  |
| Harm to Others |  | x |  |
| Self Care |  | x |  |
|  |  |  |  |
| **Diagnosis** |  |  |  |
| Indicators of Mood Disturbance |  |  | x |
| Indicators of Schizophrenia |  |  | x |
| Cognitive Problems |  |  | x |
| Anxiety |  |  | x |
| Substance use and Addictions |  |  | x |

‘Indicators of Schizophrenia’ refers to any diagnosis within the Non-affective Psychotic Disorders item according to DSM-V and any diagnosis within the category of Schizophrenia or Other Psychotic Disorders according to DSM-IV. ‘Indicators of Mood Disturbance’ refers to any diagnosis in the category of Mood Disorders in DSM-IV or any diagnosis in Mood Disorders or Bipolar Disorders categories of DSM-V. ‘Cognitive problems’ refers to any neuro-cognitive disorder according to DSM-IV/V, including dementia and Alzheimer’s Disease. ‘Anxiety’ refers to any anxiety disorder according to DSM-IV/V and post-traumatic stress disorder according to DSM-V. ‘Substance use and addictions’ refers to any substance use disorder according to the DSM-IV/V.

**Table S3.** Number of admissions per month from 2015 to 2020, average number of admissions from 2015-2019, absolute difference between 2020 and previous 5 years, and percentage difference. Negative numbers indicate decline in admission compared to the 2015-2019 period. Average and total number of admission for 2015-2019 and 2020, respectively, are presented in Figure 1A in the main text.

| **Year** | **Jan** | **Feb** | **Mar** | **Apr** | **May** | **Jun** | **Jul** | **Aug** | **Sep** | **Oct** | **Nov** | **Dec** |
| --- | --- | --- | --- | --- | --- | --- | --- | --- | --- | --- | --- | --- |
| **2015** | 3,599 | 3,253 | 3,621 | 3,413 | 3,788 | 3,479 | 3,558 | 3,620 | 3,411 | 3,648 | 3,603 | 3,330 |
| **2016** | 3,730 | 3,504 | 3,528 | 3,789 | 3,790 | 3,652 | 3,697 | 3,575 | 3,563 | 3,592 | 3,578 | 3,449 |
| **2017** | 3,775 | 3,364 | 3,789 | 3,795 | 3,777 | 3,848 | 3,767 | 3,659 | 3,634 | 3,679 | 3,656 | 3,412 |
| **2018** | 3,568 | 3,375 | 3,846 | 3,730 | 3,791 | 3,961 | 3,892 | 3,778 | 3,697 | 3,774 | 3,838 | 3,579 |
| **2019** | 3,748 | 3,396 | 3,979 | 3,666 | 3,937 | 3,909 | 3,745 | 3,900 | 3,884 | 3,801 | 3,914 | 3,686 |
| **Average** | 3,684 | 3,378.4 | 3,752.6 | 3,678.6 | 3,816.6 | 3,769.8 | 3,731.8 | 3,706.4 | 3,637.8 | 3,698.8 | 3,717.8 | 3,491.2 |
| **2020** | 3,987 | 3,765 | 3,485 | 2,548 | 3,592 | 3,625 | 3,838 | 3,893 | 3,564 | 3,807 | 3,639 | 3316 |
| **Difference** | 303.0 | 386.6 | -267.6 | -1130.6 | -224.6 | -144.8 | 106.2 | 186.6 | -73.8 | 108.2 | -78.8 | -175.2 |
| **% difference** | 8.22 | 11.44 | -7.68 | -44.37 | -6.25 | -3.99 | 2.85 | 5.03 | -2.07 | 2.92 | -2.16 | -5.28 |

**Table S4**. Results of the three logistic regression models used in the study to understand changes in the number of psychiatry admissions in Ontario, Canada during the first year of the pandemic compared to the previous five years (2015-2019). Numbers in the “N” row indicate sample sizes for all three models in each month for the COVID-19 period. Number after the hyphen in the “N” row is the sample size in the NON-COVID-19 period. NS indicates p-values equal or above 0.05, * indicates p-values less than 0.05 and equal or greater than 0.01, ** indicate values less than 0.01 and equal or greater than 0.001, and *** indicate p-values below 0.001.

| **Month** | **January** | | | **February** | | | **March** | | | **April** | | | **May** | | | **June** | | | **July** | | | **August** | | | **September** | | | **October** | | | **November** | | | **December** | | |
| --- | --- | --- | --- | --- | --- | --- | --- | --- | --- | --- | --- | --- | --- | --- | --- | --- | --- | --- | --- | --- | --- | --- | --- | --- | --- | --- | --- | --- | --- | --- | --- | --- | --- | --- | --- | --- |
| **Model** | A | B | C | A | B | C | A | B | C | A | B | C | A | B | C | A | B | C | A | B | C | A | B | C | A | B | C | A | B | C | A | B | C | A | B | C |
| **N** | 4198-18088 | | | 3737-16594 | | | 3439-18425 | | | 2531-18025 | | | 3564-18760 | | | 3589-18528 | | | 3805-18428 | | | 3856-18322 | | | 3525-17907 | | | 3762-18144 | | | 3595-18237 | | | 3280-17145 | | |
| **Demographics** |  |  |  |  |  |  |  |  |  |  |  |  |  |  |  |  |  |  |  |  |  |  |  |  |  |  |  |  |  |  |  |  |  |  |  |  |
| Age | ** | ** | ** | ns | ns | ns | ** | ** | ** | ns | ns | ns | *** | ** | *** | * | * | * | * | * | * | ns | ns | ns | ns | ns | ns | * | * | * | * | * | * | * | ** | * |
| Gender | ns | ns | ns | ns | ns | ns | ns | ns | ns | ns | ns | ns | ns | ns | ns | ns | ns | * | ns | ns | ns | ns | ns | ns | ns | ns | ns | ns | ns | ns | ns | ns | ns | ns | ns | ns |
|  |  |  |  |  |  |  |  |  |  |  |  |  |  |  |  |  |  |  |  |  |  |  |  |  |  |  |  |  |  |  |  |  |  |  |  |  |
| **Scales** |  |  |  |  |  |  |  |  |  |  |  |  |  |  |  |  |  |  |  |  |  |  |  |  |  |  |  |  |  |  |  |  |  |  |  |  |
| Positive Symptoms Scale | ns | ns |  | ns | ns |  | ns | ns |  | *** | *** |  | *** | *** |  | *** | *** |  | * | ** |  | * | *** |  | ns | ns |  | ** | *** |  | *** | ** |  | ** | * |  |
| Cognitive Performance Scale | ns | * |  | ns | ns |  | * | *** |  | ns | * |  | ns | ns |  | ns | * |  | ns | ** |  | ns | ns |  | ns | ns |  | ns | ns |  | ** | ** |  | * | *** |  |
| Depression Severity Index | ns | ns |  | ns | * |  | ** | ** |  | * | * |  | ns | ns |  | * | * |  | ns | ns |  | * | * |  | ** | ** |  | ns | ns |  | *** | *** |  | ns | ns |  |
| Aggressive Behaviour Scale | ns | ns |  | ns | ns |  | ns | ns |  | ** | ** |  | ns | ns |  | ns | ns |  | * | ** |  | * | * |  | ns | ns |  | ns | ns |  | ns | * |  | ns | * |  |
| Mania Scale | ns | ns |  | ns | ns |  | ns | ns |  | ns | ns |  | ns | ns |  | ns | ns |  | ** | ** |  | ns | ns |  | ns | ns |  | * | ns |  | ns | *** |  | ns | ns |  |
| Social Withdrawal Scale | *** | *** |  | ** | ** |  | *** | *** |  | *** | *** |  | *** | *** |  | *** | ** |  | *** | *** |  | *** | *** |  | *** | ** |  | *** | *** |  | *** | ns |  | *** | *** |  |
| Risk of Harm to Others Scale | ns |  |  | ns |  |  | ns |  |  | ns |  |  | * |  |  | ** |  |  | ns |  |  | * |  |  | ** |  |  | * |  |  | * |  |  | ns |  |  |
| Severity of Self-Harm scale | ** |  |  | ns |  |  | ns |  |  | * |  |  | ns |  |  | ns |  |  | ** |  |  | ** |  |  | ns |  |  | ns |  |  | * |  |  | ns |  |  |
| Self-Care Index | ns |  |  | ns |  |  | ns |  |  | ns |  |  | ns |  |  | ns |  |  | ** |  |  | ns |  |  | * |  |  | * |  |  | * |  |  | *** |  |  |
|  |  |  |  |  |  |  |  |  |  |  |  |  |  |  |  |  |  |  |  |  |  |  |  |  |  |  |  |  |  |  |  |  |  |  |  |  |
| **Reasons for admission** |  |  |  |  |  |  |  |  |  |  |  |  |  |  |  |  |  |  |  |  |  |  |  |  |  |  |  |  |  |  |  |  |  |  |  |  |
| Police Involvement | ** | * |  | ** | ns |  | ** | ** |  | *** | *** |  | *** | *** |  | ns | ns |  | ns | ns |  | ns | ns |  | ns | ns |  | ns | * |  | ns | ns |  | n | ns |  |
| Involuntary Admission | ns | ns |  | ns | *** |  | * | *** |  | *** | *** |  | * | * |  | *** | *** |  | *** | *** |  | *** | *** |  | ns | ns |  | ** | ** |  | ns | ns |  | * | * |  |
| Harm to Self |  | *** |  |  | ns |  |  | ns |  |  | ** |  |  | * |  |  | ns |  |  | ns |  |  | ns |  |  | ns |  |  | ns |  |  | ns |  |  | ns |  |
| Harm to Others |  | ns |  |  | ns |  |  | ns |  |  | * |  |  | ns |  |  | ns |  |  | ns |  |  | ns |  |  | ns |  |  | * |  |  | ** |  |  | ns |  |
| Self Care |  | ns |  |  | ns |  |  | ns |  |  | ns |  |  | ns |  |  | ns |  |  | ns |  |  | ns |  |  | ns |  |  | ns |  |  | * |  |  | * |  |
|  |  |  |  |  |  |  |  |  |  |  |  |  |  |  |  |  |  |  |  |  |  |  |  |  |  |  |  |  |  |  |  |  |  |  |  |  |
| **Diagnosis** |  |  |  |  |  |  |  |  |  |  |  |  |  |  |  |  |  |  |  |  |  |  |  |  |  |  |  |  |  |  |  |  |  |  |  |  |
| Mood |  |  | *** |  |  | *** |  |  | *** |  |  | *** |  |  | *** |  |  | *** |  |  | ** |  |  | ** |  |  | ns |  |  | ns |  |  | *** |  |  | *** |
| Schizophrenia |  |  | * |  |  | *** |  |  | ns |  |  | *** |  |  | * |  |  | * |  |  | ns |  |  | ns |  |  | ns |  |  | *** |  |  | ns |  |  | ns |
| Neurocognition |  |  | ns |  |  | ns |  |  | ** |  |  | ns |  |  | ** |  |  | ns |  |  | ns |  |  | ns |  |  | ns |  |  | ns |  |  | ns |  |  | ns |
| Anxiety |  |  | ns |  |  | *** |  |  | ns |  |  | *** |  |  | * |  |  | ns |  |  | ns |  |  | ns |  |  | * |  |  | ns |  |  | ns |  |  | ns |
| Substance use |  |  | ns |  |  | ns |  |  | ns |  |  | * |  |  | ** |  |  | ns |  |  | ** |  |  | ** |  |  | ** |  |  | ns |  |  | ns |  |  | ** |
| **Concordance statistics** | 0.55 | 0.55 | 0.53 | 0.55 | 0.55 | 0.54 | 0.56 | 0.56 | 0.55 | 0.59 | 0.59 | 0.56 | 0.59 | 0.58 | 0.56 | 0.57 | 0.57 | 0.54 | 0.56 | 0.56 | 0.53 | 0.56 | 0.56 | 0.53 | 0.55 | 0.55 | 0.55 | 0.57 | 0.56 | 0.55 | 0.56 | 0.56 | 0.55 | 0.58 | 0.57 | 0.54 |
